# Transcriptomics of the Rooibos (*Aspalathus linearis*) Species Complex

**DOI:** 10.3390/biotech9040019

**Published:** 2020-09-23

**Authors:** Emily Amor Stander, Wesley Williams, Yamkela Mgwatyu, Peter van Heusden, Fanie Rautenbach, Jeanine Marnewick, Marilize Le Roes-Hill, Uljana Hesse

**Affiliations:** 1 South African Medical Research Council Bioinformatics Unit, South African National Bioinformatics Institute, University of the Western Cape, Bellville 7535, South Africa; emily.amor.stander@gmail.com (E.A.S.); wesleywt@gmail.com (W.W.); yamkelamgwatyu@gmail.com (Y.M.); pvh@sanbi.ac.za (P.v.H.); 2 Institute for Microbial Biotechnology and Metagenomics, University of the Western Cape, Bellville 7535, South Africa; 3 Applied Microbial and Health Biotechnology Institute, Cape Peninsula University of Technology, Bellville 7535, South Africa; rautenbachf@cput.ac.za (F.R.); marnewickJ@cput.ac.za (J.M.); LeRoesM@cput.ac.za (M.L.R.-H.); 4 Department of Biotechnology, University of the Western Cape, Bellville 7535, South Africa

**Keywords:** rooibos, *Aspalathus linearis*, medicinal plants, non-model organism, transcriptomics, method evaluation, NGS, RNA-Seq, biochemical screening, bioinformatics

## Abstract

Rooibos (*Aspalathus linearis*), widely known as a herbal tea, is endemic to the Cape Floristic Region of South Africa (SA). It produces a wide range of phenolic compounds that have been associated with diverse health promoting properties of the plant. The species comprises several growth forms that differ in their morphology and biochemical composition, only one of which is cultivated and used commercially. Here, we established methodologies for non-invasive transcriptome research of wild-growing South African plant species, including (1) harvesting and transport of plant material suitable for RNA sequencing; (2) inexpensive, high-throughput biochemical sample screening; (3) extraction of high-quality RNA from recalcitrant, polysaccharide- and polyphenol rich plant material; and (4) biocomputational analysis of Illumina sequencing data, together with the evaluation of programs for transcriptome assembly (Trinity, IDBA-Trans, SOAPdenovo-Trans, CLC), protein prediction, as well as functional and taxonomic transcript annotation. In the process, we established a biochemically characterized sample pool from 44 distinct rooibos ecotypes (1–5 harvests) and generated four in-depth annotated transcriptomes (each comprising on average ≈86,000 transcripts) from rooibos plants that represent distinct growth forms and differ in their biochemical profiles. These resources will serve future rooibos research and plant breeding endeavours.

## 1. Introduction

Rooibos (*Aspalathus linearis*) is an indigenous South African shrub widely used to brew the popular rooibos herbal tea. The genus *Aspalathus* (Fabaceae) includes more than 270 species, most of which are endemic to the Cape Floristic Region of South Africa. Eight distinct *A. linearis* growth types have been described [1], which vary in their geographic distribution as well as in their morphological, chemical and genetic characteristics [2,3,4]. The Southern and Northern sprouters are prostrate shrublets (max 50 cm high). The Grey sprouters, Nieuwoudtville sprouters, and Wupperthal type plants are medium sized densely branched shrubs. The Red type, Black type, and Tree type plants are erect, slender bushes that can reach up to 2 m in height. The rooibos growth types can be further categorized based on their fire survival strategies: sprouters regrow after fire from an underground lignotuber while seeders are destroyed by veld fires and repopulate from seeds [3,5,6]. The commercially cultivated rooibos plants (Nortier/Rocklands type) descend from successively selected wild Red type plants originally collected from the northern parts of the Cederberg Mountains and the Pakhuis Pass areas [1]. An increasing body of literature provides scientific evidence for beneficial health effects of rooibos, including anti-inflammatory [7,8,9,10], cardioprotective [11,12,13,14,15,16], anti-diabetic [17,18,19,20], and anti-obesity [21,22,23] properties (for reviews see [24,25,26,27]). These bio-activities have been associated with the antioxidant properties of diverse phenolic compounds produced by the rooibos plants. Rooibos herbal tea is caffeine-free, low in tannins, high in volatile compounds, and rich in a unique combination of polyphenols [28]. The primary phenolic compound found in commercial unprocessed/green rooibos plant material is a C-glucosyl dihydrochalcone known as aspalathin. In commercial rooibos plants, it represents 4–12% of the plant dry weight [29,30,31]. Other major rooibos compounds include the flavones iso-orientin and orientin (the two oxidation products of aspalathin), luteolin, and chrysoeriol; as well as the flavonols rutin, hyperoside, iso-quercetin, and quercetin [32,33,34]. Wild rooibos plants were shown to have distinct chemical profiles, which differed between populations but were very similar within the same population. Some wild rooibos plants were found not to produce aspalathin at all. In these plants, orientin, iso-orientin, and rutin were found to be the main phenolic compounds [2]. The biosynthesis pathway for rooibos dihydrochalcones and flavonoids has recently been proposed [4].

The field of plant transcriptomics has been revolutionized by Next Generation Sequencing (NGS) technologies [35,36]. Transcriptomics provides a wealth of information on plant genes without prior knowledge on the underlying genome sequence, which greatly facilitates research on non-model organisms (such as rooibos). Medicinal plants usually belong to taxonomic groups that do not have high quality reference genomes [37]. In most cases, research focuses exclusively on identification of genes involved in secondary metabolite biosynthesis. On average, plants encode between 20,000 to 60,000 genes, of which only 15–25% contribute to secondary metabolite production. For these plant species, RNA-Seq is considered the method of choice to gather information on secondary metabolism associated plant genes, as whole genome analysis is considered redundant [38]. Knowledge on the genes and biosynthetic pathways involved in the production of economically important metabolites is increasingly exploited in synthetic biology and genetic engineering programs. Transcriptome data of non-model plants are already employed to optimize in vitro and in vivo biosynthesis of medicinal compounds [39,40,41], biodiesel feedstock’s [42,43,44], and essential oils [45,46,47].

South Africa is home to the unique Cape Floristic Region, where more than 70% of the plants are endemic [48]. The country has a rich traditional history in the application of diverse plant species for medicinal purposes. Yet, agricultural production systems for the nearly 3000 plant species used for traditional medicine are all but missing, and most plants are collected from the wild [49]. To promote research on the endemic medicinal plants of South Africa, this study aimed to locally establish all procedures essential for plant transcriptome analysis, including sample collection and biochemical screening methods geared at identifying interesting ecotypes from distant geographic locations, as well as laboratorial procedures and biocomputational methods for plant transcriptome analysis. The second aim of this study was to generate rooibos transcriptomes that would allow research on genes and biosynthetic pathways associated with diverse important plant traits (e.g., medicinal compound production, stress tolerance, growth form). To facilitate gene discovery, transcriptomes were sequenced from four rooibos plants that represent different growth types with distinct morphological traits and contrasting biochemical profiles (including aspalathin producers and non-producers).

## 2. Materials and Methods

### 2.1. Reagents and Materials

All solvents (analytical grade) were acquired from Merck (Kenilworth, NJ, USA). Aspalathin was obtained from Chromadex Chemicals (Los Angeles, CA, USA). Rutin, quercetin, luteolin, catechin, 4-dimethylaminocinnamaldehyde, and *p*-anisaldehyde (4-methoxybenzaldehyde) were purchased from Sigma-Aldrich (Johannesburg, South Africa), while orientin, iso-orientin, vitexin, iso-vitexin, hyperoside, and chrysoeriol were purchased from Extrasynthese (Genay, France).

### 2.2. Plant Sampling

Morphologically distinct rooibos ecotypes, including commercially farmed and wild rooibos plants representing five *A. linearis* growth forms, were sampled in the Clanwilliam (Western Cape), Wupperthal (Western Cape), and Nieuwoudtville (Northern Cape) regions in the Cederberg Mountains, South Africa. Classification of wild rooibos growth types were verified by Prof Van Wyk (University of Johannesburg) and the accurate locality was recorded by GPS. QGIS (Version 3.2.1-Bonn, https://www.qgis.org/en/site/) was used to construct the map of sampling locations using the GPS coordinates. Sampling took place in October 2016 and in February 2017. Up to 100 g of rooibos leaves and stems were collected from four sides of each plant, flash frozen in the field with liquid nitrogen, transported in a liquid nitrogen Dewar (for RNA analysis) or on dry ice (for biochemical screening), and stored at −80 °C.

### 2.3. Biochemical Analyses

To prepare plant extracts, frozen plant samples were first freeze dried and ground to a fine powder. Then, 50 mg of plant material was extracted with 5 mL methanol. All samples were mixed at 40 rpm on a Benchmark rotating mixer (Benchmark Scientific, Edison, NJ, USA) at 4 °C for 24 h.

Thin-layer chromatography was performed on all collected plant samples as described in [50]. Four plants that differed in their morphological characteristics and in their thin-layer chromatography (TLC) profiles were selected for further biochemical characterization. The October samples of these plants were analysed using HPLC. HPLC analysis of rooibos methanol extracts was conducted following the method described in [51] using a 15 cm × 4.6 mm Nucleosil 120-5C18 column (5 μM, Sigma Aldrich) on an Agilent 1200 series HPLC system coupled to a diode array detector (DAD) (Santa Clara, CA, USA). Column temperature was maintained at 21 °C. Mobile phase A was 300 μL/L trifluoracetic acid in water and mobile phase B was 300 μL/L trifluoracetic acid in methanol. A sample volume of 20 μL was injected. A constant flow rate of 1 mL/min was maintained and the gradient elution was performed as follows: 5% B for 5 min, linear increase to 80% B over 20 min, decrease to 35% B over 3 min, 35% B for 2 min, and re-equilibration to 5% B. Acquisition was set at 287 nm for aspalathin detection and 360 nm for the other polyphenols. Calibration curves were prepared from standards (aspalathin, rutin, quercetin, luteolin, catechin, orientin, iso-orientin, vitexin, iso-vitexin, hyperoside, and chrysoeriol) ranging in concentrations from 5 to 100 mg/L. The absorbance of each standard increased linearly over the concentration range. Peaks were identified by comparison to retention times and UV spectra of standards. The October samples from all four selected rooibos plants were chosen for transcriptome sequencing.

### 2.4. RNA Extraction

RNA was extracted using a modified CTAB method [52]. In brief, 250 mg of plant material was powdered in liquid nitrogen and 100 mg polyvinylpolypyrrolidone (PVPP). The powder was added to 1.5 mL RNA extraction buffer (100 mM Tris-HCL, pH 8.0; 25 mM ethylenediaminetetraacetic acid (EDTA); 2 M NaCl; 2% (*w*/*v*) polyvinylpyrrolidone (PVP); and 2% (*v*/*v*) β-mercaptoethanol), vortexed and incubated at 65 °C for 30 min with intermittent vortexing. Samples were then centrifuged for 10 min at room temperature. All centrifugation steps were performed at 16,000× *g*. The supernatant, containing nucleic acids, was extracted twice with equal volumes of chloroform:isoamyl alcohol (24:1 *v*/*v*) and centrifuged for 10 min at 4 °C. Thereafter, the supernatant was transferred to a new RNAse-free microcentrifuge tube and LiCl was added to a final concentration of 2M. RNA was precipitated overnight at 4 °C and pelleted after centrifugation at 4 °C for 1h. The RNA pellet was gently rinsed with 500 µL 70% (*v*/*v*) ethanol, air dried, and dissolved in 30 µL DEPC-treated water. RNA purity was determined spectrophotometrically at 230, 260, and 280 nm with a NanoDrop™ (ND1000, Thermo Fisher Scientific, Waltham, MA, USA) by analyzing the A260:A280 and A260:A230 ratios. Additionally, RNA concentration was measured with the Qubit Fluorometer (Thermo Fisher Scientific) and Qubit RNA BR Assay Kit (Thermo Fisher Scientific). RNA integrity was evaluated by visualization of rRNA bands on a 1.2% (*w*/*v*) denaturing agarose gel.

### 2.5. Illumina Sequencing

RNA-seq library preparation and sequencing were performed at the UKHC Genomics Core Laboratory (UK Chandler Hospital Lexington, KY, USA). Libraries were prepared with an average insert size of ~450 bp according to the Illumina TruSeq RNA Library Prep Kit v2 (Illumina, San Diego, CA, USA). Sequencing was performed on two lanes of the Illumina HiSeq 2500 platform (Illumina, San Diego, CA, USA), generating between 26.68 and 66.46 million read pairs (2 × 151 nt) according to the manufacturer’s protocols.

### 2.6. RNA-Seq Read Quality Control and Preprocessing

The supplied sequencing data had been preprocessed by the service provider, who used the Illumina bcl2FASTQ2 Conversion Software v2.20 (Illumina, San Diego, CA, USA) to perform format conversion, demultiplexing, and adapter trimming, as well as removal of PhiX reads and reads that did not contain the expected index (undetermined reads). To improve data quality, reads were further processed using Trimmomatic (v 0.36, [53]) with the following parameters: ILLUMINACLIP:TruSeq3-PE-2.fa:2:30:10 CROP:140 HEADCROP:13 MINLEN:30. Data quality was assessed before and after quality trimming using FastQC (v 0.11.4, http://www.bioinformatics.babraham.ac.uk/projects/fastqc/).

### 2.7. De novo Transcriptome Assemblies

The quality processed sequencing data from one or the four sequenced rooibos samples (plant C) was used to evaluate the performance of four transcriptome assemblers: Trinity (v 2.5.1, [54]), SOAPdenovo-Trans-127mer (v 1.03, [55]), IDBA-tran (v 1.1.1, [56]), and CLC Genomics Workbench (v 7.5.1, Qiagen). Trinity was performed using default parameters (25-mer). Only one k-mer (25-mer) was chosen for the SOAPdenovo-Trans-127mer, run using the following configuration: map_len = 32, asm_flags = 3, reverse_seq = 0. IDBA-tran was used to assemble k-mer sizes of 25–71 with a step size of 10. The CLC Genomics Workbench was used with default parameters. Transcriptome completeness was assessed using Benchmarking Universal Single-Copy Orthologs (BUSCO v0.2, [57,58]) with parameter -m tran and the embryophyta_odb9 database (accessed 2 August 2018). The read alignment rate was determined using Bowtie2 (v2.2.3, [59]) in end-to-end mode and with a “sensitive” setting.

### 2.8. Protein Prediction

TransDecoder (v5.2.0, https://github.com/TransDecoder/TransDecoder/wiki), GenemarkS-T (v 5.1, [60]) and ANGEL (v 2.4, https://github.com/PacificBiosciences/ANGEL) were used to identify candidate coding regions from the assembled transcripts. For TransDecoder, homology searches were included as open reading frame retention criteria. Transcripts with homology to known proteins were identified with (1) BlastP (v 2.2.31) against the uniref90 database (accessed 15 June 2018) with an E-value cutoff of 10^−5^, and (2) HMMER (v 3.1b2) queried against the Pfam-A database (accessed 15 June 2018). GenemarkS-T was used with a Markov chain order of 5 and default parameters. The following ANGEL scripts and parameters were employed: (1) dumb_predict.py set to “use_rev_strand”, (2) angel_make_training_set.py set to “random”, (3) angel_train.py with default parameters, and (4) angel_predict.py set to “use_rev_strand” and “output_mode = best”. 

### 2.9. Functional and Taxonomic Annotation

To identify orthologous sequence, OrthoFinder (v2.2.6, [61]) was run using the predicted proteins from the rooibos transcriptomes and the protein sequences for *Arabidopsis thaliana* (*Arabidopsis* information Resource, TAIR; accessed 25 October 2018), *Medicago truncatula* Mt4.0v2 (http://www.medicagogenome.org; [62]; accessed 25 October 2018), *Oryza sativa* V7 ([63]; accessed 25 October 2018), *Lotus japonicus* V3.0 (miyakogusa.jp 3.0 database: http://www.kazusa.or.jp/lotus/; [64]; accessed 25 October 2018), and *Lupinus angustifolius* V1.0 (http://www.lupinexpress.org/; [65]; accessed 27 January 2019]) using DIAMOND V0.9.21 [66] all-versus-all. The transcripts were further annotated using DIAMOND BLASTX against the nrNCBI database (accessed 10 August 2018) with the maximum E-value set to 1E-5. In addition, all transcripts larger than 1000 bp were assigned K-numbers (KEGG Orthology identifiers) using the web-based automatic annotation server KAAS [67] capturing the single-directional best hit (SBH analysis) via BLASTX search against the complete plant gene data set. Independently, all predicted rooibos protein sequences were also annotated with K-numbers using eggNOG-mapper (http://eggnogdb.embl.de/#/app/downloads; [68]; accessed 23 November 2018). After removing duplicated K-numbers from the KAAS and the eggNOG datasets, respectively, the resulting two lists were submitted to KEGG mapper (https://www.genome.jp/kegg/tool/map_module.html) for reconstruction of KEGG modules. Subsequently, the modules were manually filtered to represent only the 171 plant-specific modules currently available at KEGG. Finally, HMMER (V 3.1b2) was used to annotate pfam domains [69] by searching the predicted proteins against the Pfam-A database with the E-value set to 1E-5. For taxonomic assignment, transcript sequences were classified using Kraken2 v2.0.7 [70]. In addition, taxonomic ranks were assigned based on the DIAMOND BLASTX output of the transcripts using a local tool (https://github.com/pvanheus/diamond_add_taxonomy).

## 3. Results

### 3.1. Plant Sample Collection

The first aim of this study was to obtain plant material from a large number of diverse rooibos plants that would allow selection of morphologically and biochemically distinct ecotypes for subsequent transcriptome analyses. In total, 44 plants were sampled in spring (October 2016), when plants are actively growing. Of those, 37 plants survived the summer and were resampled in early autumn (February 2017), just before the harvest for herbal tea production. The plants represented five *A. linearis* growth types, including 34 Red type ecotypes (26 commercial, 4 escaped commercial, and 4 wild), as well as two Black type, two Nardouwsberg type, two Grey sprouter and four Nieuwoudtville sprouter ecotypes. The plants originated from 16 geographically distant locations in and around the Cederberg Mountains (Supplementary Figure S1). The southern-most samples were collected in the Piketberg region (153 m above sea level), and the northern-most samples originated from the wider Nieuwoudtville area (817 m above sea level). Most wild rooibos plants sampled in this study were part of a larger population of plants of the respective growth type; usually one growth type per location. Only the Black type plants were found to co-occur as single plants with other growth types.

### 3.2. Biochemical Screening of Plant Material

**Figure 1:**
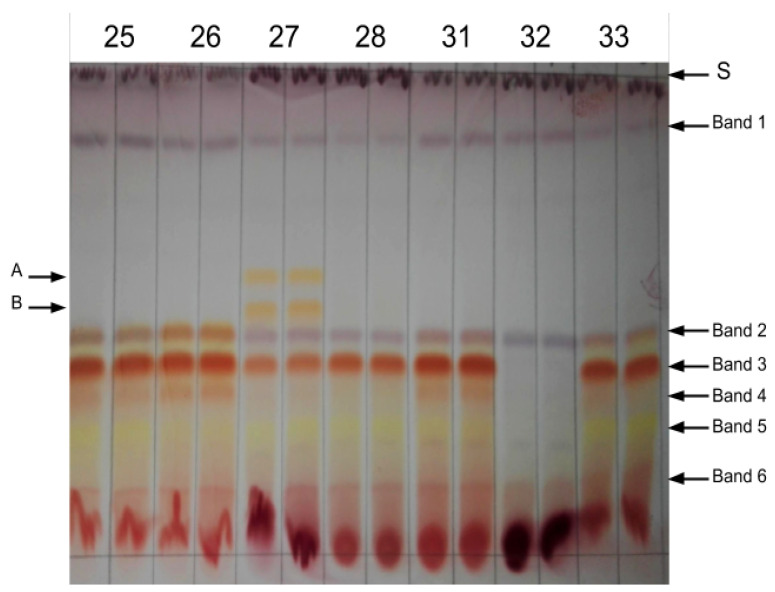
Diversity of thin-layer chromatography (TLC) banding patterns in the rooibos samples. Bands 1–6 were commonly observed in samples from commercial and wild rooibos plants; bands A and B were only found in selected plants. S: solvent front.

To visually explore the biochemical variability between the sampled rooibos plants, thin-layer chromatography (TLC) was used. Figure 1 provides a representative example of observed banding patterns. Band 3 represents aspalathin. Bands 1–6 were present in most of the commercial and wild rooibos plant samples independent of the harvest but varied in intensity depending on the TLC run. Bands A and B were only present in the autumn samples (February 2017) of the Nieuwoudtville sprouters.

For transcriptome sequencing, sample selection focused on plants with contrasting growth characteristics and distinct biochemical profiles. Based on the TLC results and the ancillary information (growth type, growth characteristics, plant health, and geographic origin), four rooibos plants were selected for comprehensive biochemical characterization using HPLC-DAD. These included one commercial Red type plant, one Black type plant, one Grey sprouter, and one Nieuwoudtville sprouter (Table 1). The selected plants varied greatly in their morphological characteristics, representing both seeders and sprouters, and showed significant differences in their biochemical profiles (Table 2). The commercial plant produced significantly more aspalathin, orientin, iso-orientin, vitexin, iso-vitexin, and hyperoside than the wild ecotypes. Aspalathin was never detected in samples of the selected Black type and Grey sprouter ecotypes. In contrast, luteolin concentrations were higher in the wild ecotype samples, and quercetin content was elevated in the Grey sprouter sample, though concentrations were still only at trace levels. All four plants were selected for transcriptome sequencing analyses.

Table 3 summarizes results pertaining to RNA extraction and Illumina sequencing of the rooibos samples. Despite high concentrations in polyphenols, the modified CTAB method was found to be effective for the extraction of high-quality RNA from the rooibos plant samples, and satisfactory RNA concentrations (46–59 ng/µL) and RNA integrity number (RIN) values (7.1–8.3) were achieved. On average, 48 Mio read pairs with an average insert size of 450 bp were generated. Sequencing quality was high, as more than 99% of the read pairs and 83.7% or the bases were retained after quality trimming, although the average read length was somewhat reduced to 126.8 nt.

### 3.3. Assessment of Transcriptome Assembly Programs

Using the reads from sample C as test data, four assembly programs, namely Trinity, IDBA-Trans, SOAPdenovo-Trans and CLC, were evaluated for their performance in assembly of rooibos RNA sequencing data (Table 4). For Trinity, two parameter settings were investigated: Trinity_all represents all assembled transcripts including different isoforms of the same gene, and Trinity_longest comprises only the longest transcripts per gene/isogroup. Trinity_all produced the highest number of transcripts (100,778 ≥ 300 bp), surpassing the other programs in all length categories. The Trinity_all assembly also showed the highest read realignment rate (97%) and the highest number of matches to the 1440 plant BUSCOs, identifying in total 92%. However, when this dataset was filtered for the longest transcript per isoform (Trinity_longest), half of the total number of transcripts (including two-thirds of the transcripts >1 kb) were lost, as they represented different versions of the same gene/isogroup. Transcript filtering resulted in a very low proportion of duplicated BUSCO matches in the Trinity_longest assembly (2.5%), but also reduced informative content and assembly accuracy of the transcriptome (the proportion of total BUSCO matches dropped to 86% and the number of fragmented BUSCO matches doubled). IDBA-Trans assembled a substantially lower number of transcripts than Trinity_all, specifically in the smaller length categories (≤1 kb). However, the number of transcripts longer than 1 kb (≈41,000) was comparable to the one obtained with Trinity_all (≈44,000), and 90% of the plant BUSCOs were reassembled. The IDBA-Trans assembly had the highest proportion of duplicated BUSCOs (indicating redundancy), as well as the lowest number of fragmented BUSCOs and the highest proportion of transcripts that matched a BUSCO (indicating assembly accuracy). SOAPdenovo-Trans produced the lowest total number of transcripts (50,503), approximately half of which were longer than 1 kb. It was the only assembler that produced transcripts longer than 10 kb. However, the concordant read alignment rate was very low (58%) and the proportions of fragmented (16%) and missing (13%) BUSCOs were high, indicating low assembly accuracy. CLC assembled a large number of smaller transcripts (≈45,000 ≤ 1 kb); only 14,169 transcripts were longer than 1 kb. Consequently, this assembly matched the least number of BUSCOs, and many of the transcripts that did match a BUSCO were fragmented. The above analyses indicated that IDBA-Trans produced a comprehensive, comparatively accurate assembly with the lowest proportion of short and/or misassembled fragments. It was therefore chosen for subsequent assembly of the remaining rooibos transcriptomes.

### 3.4. Assessment of ORF Prediction Tools

Diverse downstream annotation operations can be sped up or only be conducted when using protein sequences (e.g., BlastX vs. BlastP; pfam annotations). To identify the most suitable program for prediction of open reading frames (ORFs) on transcripts, the IDBA-Trans assembly of sample C was used to compare performances of ANGEL, GenemarkS-T, and TransDecoder (Table 5). The highest number of predicted ORFs were obtained when using ANGEL, which identified ORFs on 93% of the 76,784 transcripts. GenemarkS-T and TransDecoder predicted ORFs on only 71% of the sequences. While ANGEL and GenemarkS-T could predict multiple ORFs per transcript, TransDecoder predicted only one. Comparisons with the BUSCO annotations of the transcriptome showed that ORF prediction resulted in a slight reduction of total BUSCO hits, ranging between 7 (ANGLE) and 19 (GenemarkS-T) missed BUSCO sequences. For complete BUSCOs, ORF prediction increased the number of single-copy BUSCOs and reduced the number of duplicated BUSCOs, indicating that a number of nucleotide sequences that had matched a BUSCO were misassembled and did not encode ORFs. Transcript misassemblies leading to ORF truncations could also explain the somewhat higher numbers of fragmented BUSCOs in the predicted ORF datasets. Based on the above results, ANGEL was chosen for downstream analyses.

### 3.5. Rooibos Transcriptome Assemblies

IDBA-Trans was used for de novo assembly of the four sequenced transcriptomes (Table 6). Depending on the sample, the program generated between 76,784 and 96,865 transcripts ≥300 bp. Approximately 50% of the transcripts were longer than 1 kb, and few transcripts were longer than 5 kb. Read usage across all transcriptomes was >66%, with 51.94–75.86% of reads aligning concordantly to the transcripts. ORFs were predicted on 93–98% of the transcripts. On average, the transcriptomes comprised 1246 complete and 59 fragmented BUSCOs. RIN values, but not read numbers, appeared to have an effect on BUSCO statistics. Across the four transcriptomes, we observed that lower RIN values were associated with higher numbers of missed and fragmented BUSCOs. We also noted that the number of BUSCO hits did not substantially change if read numbers were increased by 2×–2.5×, as seen for sample C vs. samples A and B.

### 3.6. Rooibos Transcriptome Annotation: Comparative Genomics

**Figure 2:**
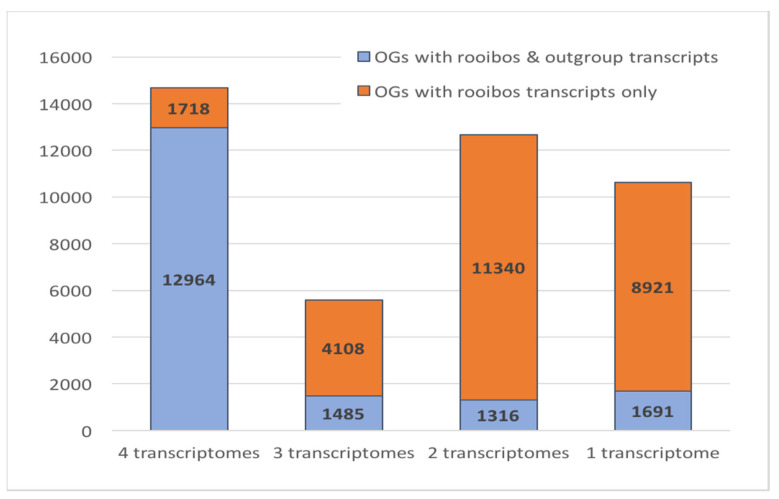
Distribution of rooibos-specific and general plant orthologous groups (OGs) across the transcriptomes.

Analysis of orthologous relationships between proteins from different plant species allows transfer of functional annotations to new protein sequences. In this study, we compared the four rooibos transcriptomes to the proteomes of three legumes (*L. angustifolius, L. japonicus,* and *M. truncatula)* and the model plants *A. thaliana* and *O. sativa* (Supplementary Table S1). Of the 331,195 rooibos proteins, 85% were assigned to orthologous groups (OGs), i.e., were partnered with at least one other sequence from the investigated datasets. In total, 43,543 OGs included rooibos protein sequences. The number of OGs per rooibos transcriptome ranged between 26,334 and 29,535 OGs. Of those, 14,682 OGs appear to represent gene families that are highly conserved among the rooibos growth types as they included sequences from all four transcriptomes (Figure 2). In this subset, the proportion of rooibos-specific OGs that did not include proteins from any of the investigated outgroup species (*L. angustifolius*, *L. japonicus*, *M. truncatula*, *A. thaliana,* and/or *O. sativa*) was only 12%. This was in stark contrast to OGs where one or more rooibos transcriptomes were missing; in these subsets, the proportion of rooibos-specific OGs ranged between 73% and 90%. While it is possible that some of the unassigned rooibos proteins that did not group with any other sequence may represent ecotype- or growth form-specific genes, transcript misassembly or fragmentation is a more likely cause for low sequence similarity to other proteins.

### 3.7. Rooibos Transcriptome Annotation: Taxonomic Transcript Classification

The taxonomic annotations from Kraken2 and NCBI (NR) allowed prediction of the organism from which the transcripts were derived (Table 7). Kraken2 yielded 17,604 more taxonomic transcript annotations than sequence comparisons to the NCBI (NR) database, but the latter permitted deeper taxonomic classification. Both analyses confirmed that the absolute majority of the annotated transcripts (94.9–99.9%) were of plant origin, most matching proteins from leguminous plants. The transcriptomes from the wild rooibos plants (B, C, and D) also contained a considerable number of fungal transcripts. All three samples harbored sequences from *Dothistroma septosporum*, *Ascochyta rabiei,* and *Alternaria alternata*. In addition, the transcriptomes from plants B and C shared a considerable number of sequences that matched *Baudoinia panamericana*, *Hortaea werneckii,* and *Elsinoe australis*. For several transcripts, Kraken2 annotations indicated bacterial origin, but this was not confirmed by the DIAMOND-NCBI(NR) analysis, which annotated most of them as plant transcripts.

### 3.8. Rooibos Transcriptome Annotation: Functional Annotation

Functional annotations for the rooibos transcript and protein sequences are summarized in Table 8. Across the four rooibos transcriptomes, functional annotations were generated for 256,962 transcripts (74%) and 209,529 predicted proteins (63%). Most annotations were obtained through the DIAMOND-NCBI (NR) analysis, as 72–79% of the rooibos transcripts larger than 300 bp matched a protein. The KEGG BLAST server KAAS, which only analyses sequences larger than 1 kb, provided K-numbers and enzyme identifiers for 23% and 13% of all transcripts, respectively. EggNOG yielded functional annotations for 63% of the protein sequences and K-numbers for 32% of the protein sequences, substantially outperforming KAAS in the assignment of K-numbers (both, total and unique). Furthermore, half of the predicted rooibos proteins had Pfam-A annotations.

**Figure 3:**
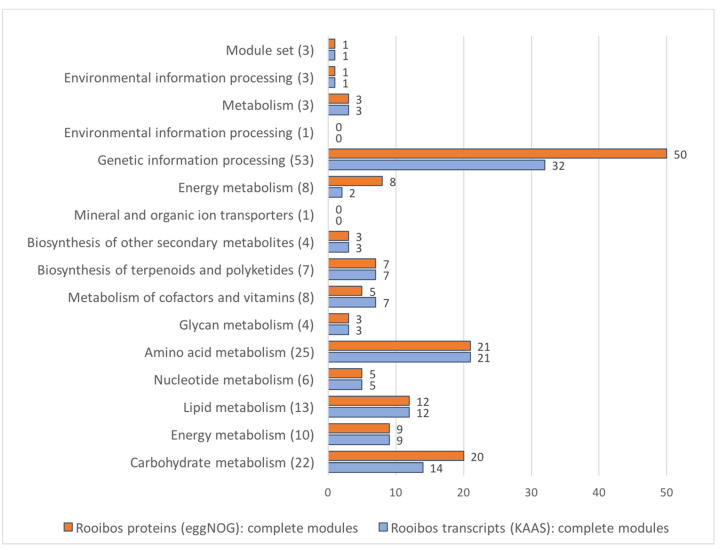
KAAS vs. eggNOG-mapper in terms of annotated complete plant-specific KEGG modules covered. The number of KEGG database plant-specific modules that were annotated to completion by rooibos transcripts using KAAS (blue) or rooibos proteins using eggNOG (orange) are compared. The total number of each plant specific module in the KEGG database is depicted in brackets.

The K-numbers provided by KAAS and eggNOG were used to link the transcripts and proteins to KEGG modules. Currently, the KEGG database contains 171 plant-specific modules (PSMs). When combining all rooibos data, 150 PSMs were annotated to completion (120 by KAAS and 148 using eggNOG; Figure 3). When compared to eggNOG, KAAS failed to complete annotation of six carbohydrate metabolism, six energy metabolism, and 18 genetic information processing modules, but completed annotation of two modules associated with metabolism of cofactors and vitamins that were annotated incompletely by eggNOG. Both annotation procedures missed the same six plant-specific modules. Four of these have only been annotated in algae (M00338 M00185 M00616 M00741) and one is specific to monocotyledonous plants (M00369). A major signature module that appears to be missing in both annotation sets of the rooibos transcriptomes was the biosynthetic pathway for “Oxygenic photosynthesis in plants and cyanobacteria” (M00611). This pathway is present in 39% of the plant species currently stored in the KEGG database, including model organisms such as *A. thaliana* and *O. sativa* as well as in legumes, such as *Glycine max, L. japonicas,* and *Phaseolus vulgaris*. 

The eggNOG annotation set contained three complete non-plant modules (M00133, M00393, and M00516), one of which (M00516: SLN1-YPD1-SSK1/SKN7 (osmosensing) two-component regulatory system) is specific to fungi. This module was annotated to completion in transcriptome B, the dataset that had the highest number of fungal transcripts. However, the specific transcripts that encoded these enzymes were all classified as plant transcripts by both, DIAMOND-NCBI (NR) and Kraken2. The KAAS annotation set contained only one complete non-plant module: M00116 is specific to bacteria and is associated with the biosynthesis of menaquinone through the chorismate => menaquinol pathway. Only the transcriptomes B and D contained the complete module. Again, the transcripts that encoded these enzymes were classified as plant transcripts by DIAMOND-NCBI (NR) and Kraken2.

## 4. Discussion

### 4.1. High-Throughput Screening of Wild Plants for Transcriptome Analyses

The first aim of the study was to identify rooibos plants of interest for transcriptome sequencing. To study rooibos-specific biosynthetic pathways involved in the production of medicinally/bio-active polyphenols (such as aspalathin), it was essential to find plants with contrasting biochemical profiles. So far, few studies have focused on the variability of polyphenol profiles in rooibos plants [2,4]. Aspalathin non-producers have only been reported among the wild growing Black type plants and Grey sprouters [2]. The sampling pool established in this study therefore comprised 44 rooibos ecotypes from distant geographical locations that span the complete rooibos production region and included 26 commercial plants and 18 wild rooibos ecotypes representing five rooibos growth types.

Biochemical screening of plant samples for specific compounds of interest can represent a cost-limiting factor, since many of these compounds can only be detected using expensive technologies such as HPLC, Near Infrared Spectroscopy (NIS) or Raman Spectroscopy (RS). In this study, high-throughput biochemical screening was completed using thin layer chromatography (TLC) analyses that permitted detection of aspalathin and identification of plant-specific fingerprints [50]. TLC is a simple and easily scalable method for detection of diverse compounds in plant extracts, herbal products, and foods [66]. When paired with chromatogram densitometry or image analysis it even permits compound quantification, providing a cost-effective alternative to other detection methods (e.g., HPLC, NIS, RS). TLC has been extensively used in plant research to generate biochemical fingerprints that differentiate between species, subspecies, and even ecotypes [71]. It has also been found useful for screening samples for marker compounds, that facilitate species authentication and quality control of plant and herbal product samples [72].

Based on morphological plant characteristics and biochemical profiles, four rooibos plants were selected for transcriptome sequencing. The selected plants represented different growth types (Red type, Black type, Nieuwoudtville sprouter, and Grey sprouter), and therefore included rooibos plants that differ in their drought adaptation and fire survival strategies (seeders and sprouters). The Nieuwoudtville sprouter is an extreme example; it was never observed to produce flowers (as monitored each spring and autumn for a period of three years). Two of the selected plants did not produce aspalathin (the Black type and the Grey spouter). The commercial rooibos plant displayed a typical “super plant” phenotype; it was one of the largest plants in the field on both harvest occasions, having survived the dry summer months unaffected, and consistently produced high amounts of polyphenols.

### 4.2. Establishing Biocomputational Procedures for Non-Model Plant Transcriptome Analyses

Plant transcriptomics is on the rise as Next Generation Sequencing technologies provide cost-effective means to access this exceptional genomic resource. However, biocomputational procedures must be adapted to address specific problems associated with plant transcriptome sequencing data. Compared to other eukaryotes, plants have larger gene families, larger amounts of highly expressed transposable elements [55] and highly variable expression levels, found to span up to five orders of magnitude [73,74]. Therefore, recent scientific publications focus on the optimization of strategies for the assembly and annotation of plant transcriptomes [75,76,77].

In 2017, Wang and Gribskov [75] investigated the effectiveness of eight transcriptome assembly programs (BinPacker, Bridger, IDBA-Trans, Oases-Velvet, SOAPdenovo-Trans, SSP, Trans-ABySS, and Trinity) for their ability to accurately reassemble RNA-seq data from the model plant *A. thaliana*. The authors identified SOAPdenovo-Trans as the most suitable assembly program based on the base coverages of the genome (portion of bases from the reference genome covered by the transcriptome) and the transcriptome (portion of bases on the transcriptome covered by the reference genome), as well as the read realignment rates. Assembly success can be affected by a diverse number of factors, such as tissue type, RNA quality, sequencing platform, sequencing depth, and data pre-processing, to name but a few [78,79]. It is therefore advisable to re-evaluate assembler performance for new plant RNA sequencing data, in particular if it was derived from a non-model organism where the genome sequence is not available for transcript assembly verification. In light of the increasing number of sequencing projects that focus on non-model organisms, new metrics for quality assessment of transcriptome assemblies are essential. A rational approach is the analysis of newly assembled transcriptomes for sequences that show significant similarity to well described, highly conserved genes. Benchmarking Universal Single-Copy Orthologs (BUSCO; [57]) aims to standardize this metric by constructing a reference database composed of single-copy orthologs that are present in at least 90% of plant species from OrthoDB (www.orthodb.org).

In this study, five assemblers were assessed for their ability to correctly reconstruct a rooibos transcriptome. Trinity can be run with two settings either providing multiple isoforms per isogroup or filtering for the longest isoform per isogroup. The unfiltered Trinity_all assembly produced the highest number of transcripts (>100,000) and consequently had the highest rate of re-aligned reads (97%). It also had the highest number of BUSCO hits; however, a large proportion of the transcripts (43%) was duplicated. Filtering for the longest transcript per isogroup reduced the dataset by half, effectively removing duplicates (the proportion of duplicated BUSCOs was reduced to 3%). However, the longest transcript did not always represent the best assembled isoform per isogroup as indicated by substantial reductions of BUSCO hits. IDBA-Trans produced the second-largest transcriptome (~77,000 transcripts), which matched a similar number of BUSCOs as the transcripts from the Trinity_all assembly despite the substantially lower number of transcripts. The high proportion of duplicated BUSCOs (66%) indicated that the IDBA-Trans dataset was redundant (i.e., contained multiple transcripts per gene). Since plant genes often encode multiple splice variants [80], this dataset represented a more realistic reconstruction of the rooibos transcriptome than the Trinity_longest assembly. SOAPdenovo-Trans produced fewer transcripts (~50,000) tending to assemble longer sequences. It was the only assembler that produced two transcripts >10 kb (which were assembled correctly both matching the SYD-like chromatin structure-remodeling complex protein from *L. angustifolius*). However, this assembly was less informative and more fragmented than the IDBA-Trans assembly as indicated by the BUSCO analysis results. CLC was outperformed by the other assemblers in all assessed parameters. The results of this study therefore contrasted with the findings by Wang and Gribskov (2017) [75] that SOAPdenovo-Trans is the most suitable assembler for plant data analyses, indicating that IDBA-Trans may be a better alternative for some datasets. Identification of ORFs and subsequent protein prediction facilitates functional annotation of transcripts permitting the use of protein-based annotation programs such as eggNOG-mapper and HMMER-Pfam. Therefore, three protein prediction algorithms (ANGEL GenemarkS-T and TransDecoder) were compared for their accuracy in ORF prediction using the IDBA-Trans assembly of the transcriptome from plant C. The highest number of ORFs was identified by ANGEL resulting in somewhat better BUSCO annotations. It was therefore considered most suitable for ORF and protein prediction analyses.

### 4.3. The Rooibos Transcriptomes

The four transcriptomes yielded on average approximately 86,000 transcripts per transcriptome. Over 90% of these transcripts were predicted to encode proteins. Combined, these transcriptomes matched over 90% of the 1440 plant BUSCO sequences indicating that the datasets are comprehensive in terms of functionally meaningful sequences. Orthology analyses, which included the predicted proteins from the four rooibos transcriptomes and the complete protein datasets from the sequenced genomes of three legumes (*L. angustifolius*, *L. japonicus*, and *M. truncatula*) as well as distantly related model plants (*A. thaliana* and *O. sativa*) supported this assumption. The number of OGs shared between the protein datasets from already sequenced plant genomes (10,685–13,719 OGs) was comparable to the number of OGs shared between the respective rooibos transcriptomes and the other plant species (10,873–13,546 OGs), implying that most common plant gene families were present in the rooibos datasets. Surprisingly, the rooibos transcriptomes shared the highest numbers of OGs with the protein dataset from *L. japonicas* although *A. linearis* is much closer related to *L. angustifolius*. However, the protein dataset from *L. angustifolius* was notably smaller than the ones from the other two legumes, which may explain the smaller number of OGs shared with the rooibos transcriptomes. *M. truncatula* is taxonomically just as distantly related to rooibos as *L. japonicus* and the protein dataset was larger, yet on average it shared approximately 107 fewer OGs with the rooibos datasets. First screens indicated that many of the OGs missing in *M. truncatula* appear to encode proteins involved in signal transduction. Future investigations are necessary to explain this observation. Only 15% of the rooibos proteins (av. 12,828 per transcriptome) could not be assigned to an OG, i.e., they did not have counterparts in the rooibos transcriptomes or in any of the protein datasets from sequenced plant genomes. This number is comparable to results obtained for other recently sequenced non-model plant datasets (e.g., ~24,000 in Zingiberales: [81]; 15,296 in geophytes: [82]). These sequences may represent rooibos-specific genes, but also chimeras and/or truncated ORFs.

Taxonomic classification of transcripts is often omitted in transcriptome analyses. However, it represents an essential procedure if one wants to avoid contamination of databases and subsequent propagation of misannotations. In this study two approaches were investigated: Kraken2 and DIAMOND-searches against NCBI(NR). For the four rooibos transcriptomes, Kraken2 provided taxonomic affiliations for a higher number of transcripts, annotating between 77% and 82% of the transcript sequences, as compared to DIAMOND-NCBI(NR), which classified 69% to 78% of the transcripts. Kraken2 is a “fast” sequence analysis program primarily designed for taxonomic classification of sequences in metagenomic datasets, which often consist of millions to billions of reads. Therefore, there is a trade-off between sensitivity, accuracy, performance, speed, and computational requirements. Although these types of programs are useful in performing “first-pass” taxonomic analyses it is crucial that their assignments are validated using BLAST-based procedures [83]. The absolute majority of the transcripts were of plant origin, most hitting legume sequences as identified by DIAMOND-NCBI(NR) analysis. However, bacterial and fungal sequences were also predicted. In this study, bacterial sequences should have been very rare if not completely absent from the datasets: prior to sequencing, RNA samples had been enriched for mRNA using oligo(dT) beads; bacterial RNA is not polyadenylated and should not have been captured. However, Kraken2 annotated 1564 (0.5%) of the transcripts as bacterial. For most of them, the DIAMOND-NCBI(NR) annotations indicated plant origin, suggesting that in these cases the Kraken2 annotations were incorrect. This bias towards bacterial sequence annotations may be associated with the Kraken2 database; it contains only the NCBI(RefSeq) proteins derived from fully sequenced genomes. Since the number of sequenced bacterial genomes is far higher than the numbers of fungal and plant genomes, annotation biases are inevitable. However, even the DIAMOND-NCBI(NR) analysis indicated bacterial origin for 172 transcripts (0.05%). Further investigations showed that some of these transcripts encoded phage proteins (Replication-associated protein A) and transposases, which are subject to horizontal gene transfer and therefore difficult to taxonomically classify. Some transcripts encoded cell wall associated hydrolases and acyl-CoA-binding proteins. These enzymes can be found in both plants and bacteria, and misclassification due to sequence similarity is likely. For other transcripts, the annotation was based on the shorter ORF, and the longer ORF of the same transcript would encode a plant protein. We conclude that while it is not impossible that some of the above transcripts originated from bacteria, the absolute majority of them were misclassified. Notable numbers of fungal transcripts were identified only in the transcriptomes from the wild rooibos plants B, C, and D although all appeared healthy at harvest time. The highest proportion of fungal transcripts were found in the transcriptome of plant B (3% as per DIAMOND-NCBI(NR) analysis). Most of these transcripts matched the plant pathogenic fungi *D. septosporum*, known to cause red band needle blight in conifers [84], and *A. rabiei*, the causative agent of ascochyta blight in diverse plant species [85]. In transcriptome D the majority of fungal transcripts apparently originated from *A. alternata,* another well-known plant pathogen. These transcripts indicate the presence of low levels of fungal infections in the wild rooibos plants. Commercial rooibos plants are often treated with fungicides and pesticides which may explain the near absence of fungal transcripts in the investigated transcriptome from plant A. Of interest was also the finding that plants B and C, though separated by a distance of 37 km, both appeared to host the same two black yeast species. *B. panamericana* has not yet been described in plants but rather as a fungus that grows on walls, roof tiles, and on vegetation around distilleries, giving it the more common name “whisky fungus” [86]. *H. werneckii* is a halotolerant yeast that was recently found to endophytically colonize the Chinese medicinal plant *Aegiceras comiculatum* [87]. These findings emphasize the power of transcriptome analysis as a tool for investigating symbiotic relationships with diverse microorganisms that populate plants, provided that database biases are taken into consideration.

Up to 256,962 rooibos transcripts (74%) and 209,529 (63%) predicted protein sequences could be functionally annotated, showing significant matches to sequences and/or protein domain profiles in the NCBI(NR), KEGG, eggNOG, and/or Pfam-A databases. Most annotations were derived through the DIAMOND-NCBI(NR) analysis (74%). Though informative, these annotations are difficult to summarize. KEGG and eggNOG database searches provided K-numbers which linked the rooibos transcripts to biosynthetic pathway modules (KEGG modules) and allowed assignment of enzyme numbers and gene ontology terms. The eggNOG-mapper provided more plant-specific K-number assignments than the KEGG Automatic Annotation Server, KAAS (4163 versus 3736 K-numbers). The KAAS online server accepts only a limited number of sequences requiring batch-analysis; therefore, only transcripts larger than 1 kb were selected for analyses. This not only reduced the total number of K-numbers identified by KAAS but also the number of unique K-numbers for the rooibos transcriptome datasets.

KEGG represents biosynthetic pathways as separate building blocks. The smallest units are the enzymes that have unique identifiers—the K-numbers. Enzymes that catalyze a specific reaction are grouped into blocks, which themselves are arranged into biosynthetic pathways—the KEGG pathway modules. A given KEGG pathway module is considered complete if all blocks are represented by at least one K-number. KEGG also provides a similar hierarchical architecture for structural complexes, functional sets, and signature modules. Currently the KEGG database contains 171 plant-specific KEGG modules. The KAAS annotations completed 70%, and the eggNOG-mapper annotations completed 87% of the plant-specific modules. Only six plant-specific KEGG modules were missing in both datasets. Four of the missing modules are specific to algae and would therefore not be expected to be present in rooibos. One important plant-specific module missing in both datasets was the pathway module M00611 (oxygenic photosynthesis in plants and cyanobacteria), which is present in 39% of all plant KEGG entries. This module comprises three submodules: photosystem II (M00161), photosystem I (M00163), and Reductive pentose phosphate cycle (Calvin cycle) (M00165). It is important to note that separately these three modules are NOT specific to plants but are also found in protists and bacteria. However, these three modules together form the supermodule M00161, which is plant-specific. Upon further inspection, it was found that both the KAAS and the eggNOG datasets contained the complete submodule M00165. The KAAS dataset lacked several K-numbers from modules M00161 and M00163 (containing five of the six K-numbers for photosystem I and four of the six K-numbers for photosystem II). The three missing K-numbers in the KAAS dataset were entries for proteins that are smaller than 354 amino acids (psbD: 353 amino acids psbF: 39 amino acids psaC: 81 amino acids). Therefore, their transcript sequences did not pass the minimum transcript length cutoff (1kb threshold) set for KAAS server analyses. The eggNOG dataset was more complete lacking only one K-number (k02708) for photosystem II, which may have been missed due to low transcript expression levels or transcript misassembly. These results exemplify that a sequence length cutoff of 1kb is not suitable for functional annotation of plant transcriptomes and that KEGG annotations should preferably be conducted using eggNOG on a local cluster.

**Figure 4:**
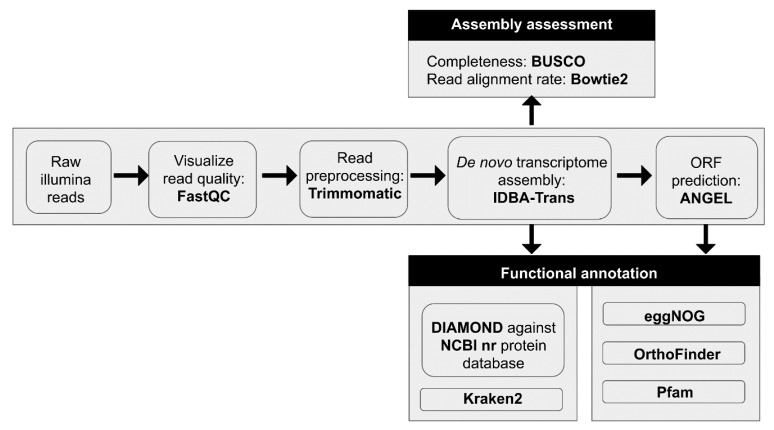
Established biocomputational pipeline for high-throughput comprehensive plant transcriptome analysis.

The above analyses allowed the establishment of a biocomputational pipeline for comprehensive, high-throughput plant transcriptome analysis (Figure 4).

## 5. Conclusions

This study represents a first undertaking to investigate the genomic background of an endemic South African medicinal plant species. The rooibos transcriptomes provide a first extensive dataset that can be mined for genes involved in diverse biosynthetic pathways of interest: medicinal compound production, stress response, morphological characteristics, and plant-microbe interactions to name but a few. The methods established for biocomputational sequencing data analyses are applicable to a wide range of other non-model plant species.

## Figures and Tables

**Table 1:** Botanical and growth characteristics of rooibos plants selected for transcriptome analyses.

Plant	Growth Type	Seeder/Sprouter	Growth Form	Location
**A**	Red type (commercial)	seeder	upright, densely branched bush	S 031° 43′ 18′′ E 019° 07′ 32′′
**B**	Nieuwoudtville sprouter	sprouter	low-growing, densely branched bush	S 031° 45′ 48′′ E 019° 07′ 54′′
**C**	Black type	seeder	tall, slender shrub	S 031° 59′ 21′′ E 018° 50′ 35′′
**D**	Grey sprouter	sprouter	upright, sparsely branched bush	S 032° 37′ 17′′ E 019° 03′ 24′′

**Table 2:** Biochemical characterization of rooibos plants selected for transcriptome analyses (October 2016 samples).

Plant	Aspalathin	Orientin	Iso-Orientin	Iso-Vitexin	Vitexin	Hyperoside	Luteolin	Quercetin
**A**	66.52 ± 0.68 ^a^	4.70 ±0.19 ^a^	6.94 ± 0.05 ^a^	2.18 ± 0.07 ^a^	1.98 ± 0.10 ^a^	2.06 ± 0.09 ^a^	0.01 ± 0.00 ^a^	0.01 ± 0.00 ^a^
**B**	9.24 ± 0.17 ^b^	1.56 ± 0.04 ^c^	1.50 ± 0.01 ^c^	0.17 ± 0.00 ^c^	0.49 ± 0.01 ^b^	0.41 ± 0.01 ^b^	0.21 ± 0.00 ^b^	0.01 ± 0.00 ^a^
**C**	0.00 ± 0.00 ^c^	1.82 ± 0.08 ^c^	1.71 ± 0.03 ^c^	0.17 ± 0.00 ^c^	0.23 ± 0.01 ^c^	0.32 ± 0.01 ^b,c^	0.59 ± 0.01 ^c^	0.01 ± 0.00 ^a^
**D**	0.00 ± 0.00 ^c^	2.29 ± 0.06 ^b^	2.85 ± 0.08 ^b^	0.69 ± 0.01 ^b^	0.25 ± 0.00 ^c^	0.17 ± 0.00 ^c^	0.58 ± 0.01 ^c^	0.05 ± 0.00 ^b^

* Concentrations of major flavonoids are provided in mg/100 g dry weight ± STD (*n* = 3). Biochemical analyses were conducted using HPLC-DAD. Different letters indicate significant differences as verified using the Tukey test.

**Table 3:** Summary of the sequencing data generated from the October 2017 samples of four rooibos ecotypes.

Plant	A	B	C	D
**RNA yield (ng/µL)**	53.0	59.0	58.0	46.0
**RNA integrity number (RIN)**	8.3	7.1	7.9	8.1
**Library insert size (bp)**	437	448	479	452
**# read pairs (in Mio)**	54.7	66.5	26.7	44.1
**# read pairs after quality processing (in Mio)**	54.6	65.8	26.5	43.8
**% read pairs remained after quality processing**	99.9	99.1	99.2	99.2
**% bases remained after quality processing**	83.6	83.7	83.8	83.7
**Length after trimming (bp)**	30–127	30–127	30–127	30–127

**Table 4:** Comparison of four different de novo transcriptome assemblies, assembled from the processed reads from sample C.

	Trinity_all	Trinity_longest	IDBA_Trans	SOAPdenovo_Trans	CLC
**Assembler running time (h)**	22	22	4	1	4
**# of Transcripts (≥300 nt)**	100,778	53,363	76,784	50,503	59,716
**300–500 bp:**	28,701	22,049	15,941	11,203	27,416
**501–1000 bp:**	27,747	14,995	19,885	13,139	18,131
**1001–5000 bp:**	43,922	16,145	40,701	25,795	14,046
**5001–10,000 bp:**	408	174	257	364	123
**>10,000 bp:**	0	0	0	2	0
**Overall read alignment rate (%)**	97.0	82.9	89.1	76.8	78.7
**Read pairs aligned concordantly (%)**	82.0	66.0	75.9	58.1	59.8
**Complete BUSCOs (C)**	1258	1092	1230	1019	923
**Single-copy BUSCOs (S)**	721	1061	374	825	870
**Duplicated BUSCOs (D)**	537	31	856	194	53
**Fragmented BUSCOs (F)**	73	152	59	229	244
**Missing BUSCOs (M)**	109	196	151	192	273
**# of transcripts that hit a BUSCO**	2065	1278	2561	1477	1223
**% of transcripts that hit a BUSCO**	2.0	2.4	3.3	2.9	2.0

**Table 5:** Comparison of gene-finding algorithms GenemarkS-T, TransDecoder, and Angel.

	Transcriptome (76,784)	Angel	GenemarkS-T	TransDecoder
**# of predicted proteins**	-	74,767	58,284	54,205
**# of transcripts with ORFs**	-	71,791	54,754	54,205
**ORFs/transcript (mean)**	-	1.04 ± 0.21	1.06 ± 0.26	1.00 ± 0.00
**Complete BUSCOs**	1230	1211	1202	1200
**Single-copy BUSCOs**	374	396	394	390
**Duplicated BUSCOs**	856	815	808	810
**Fragmented BUSCOs**	59	71	68	74
**Missing BUSCOs**	151	158	170	166

**Table 6:** Rooibos transcriptomes.

	A	B	C	D
**Total Mbps**	121.87	122.11	98.02	102.83
**Transcripts**	91,171	96,865	76,784	80,456
**300–500 bp**	20,986	24,955	15,941	18,460
**501–1000 bp**	22,161	24,463	19,885	20,767
**1001–5000 bp**	47,231	46,736	40,701	40,674
**5001–10 000 bp**	793	707	257	547
**>10,000 bp**	0	4	0	8
**Predicted ORFs**	85,234	91,301	75,426	79,234
**Overall read alignment rate (%)**	77.11	66.75	89.05	81.11
**Read pairs aligned concordantly ≥ 1× (%)**	60.63	51.94	75.86	63.66
**Read pairs aligned discordantly (%)**	7.63	5.60	8.74	8.57
**RIN**	8.3	7.1	7.9	8.1
**Complete BUSCOs (C)**	1291	1221	1230	1242
**Complete and single-copy BUSCOs (S)**	383	335	374	391
**Complete and duplicated BUSCOs (D)**	908	886	856	851
**Fragmented BUSCOs (F)**	48	65	59	62
**Missing BUSCOs (M)**	101	154	151	136

**Table 7:** Taxonomic transcript annotations using DIAMOND-NCBI(NR) and Kraken2.

	A (91,171)		B (96,865)		C (76,784)		D (80,456)	
**NCBI Nr Taxonomic category**	**Transcripts**	**%**	**Transcripts**	**%**	**Transcripts**	**%**	**Transcripts**	**%**
**Fabaceae**	62,368	68.41	63,097	65.14	57,558	74.96	57,878	71.94
**Other Plants**	2905	3.19	3036	3.13	2324	3.03	2566	3.19
**Fungi**	4	0	3140	3.24	661	0.86	664	0.83
**Bacteria**	28	0.03	38	0.04	24	0.03	82	0.1
**Viruses**	15	0.02	14	0.01	4	0.01	8	0.01
**Other Eukaryotes**	16	0.02	333	0.34	199	0.26	0	0
**Total:**	65,336	71.66	69,658	71.91	60,770	79.14	61,198	76.06
**Kraken2 classification**	**transcripts**	**%**	**transcripts**	**%**	**transcripts**	**%**	**transcripts**	**%**
**Plant**	72,016	78.99	72,771	75.13	62,359	81.21	63,673	79.14
**Bacteria**	387	0.42	493	0.51	340	0.44	344	0.43
**Fungi**	78	0.09	1467	1.51	345	0.45	293	0.36
**Total:**	72,481	79.5	74,731	77.15	63,044	82.11	64,310	79.93

**Table 8:** Number of transcript and protein sequences annotated in each rooibos transcriptome.

	A	B	C	D
**Total transcripts (>300 bp)**	**91,171**	**96,865**	**76,784**	**80,456**
*Transcripts > 300 bp annotated using NCBI(NR)*	65,336	69,658	60,770	61,198
**Total transcripts (>1000 bp)**	**48,024**	**47,447**	**40,958**	**40,958**
*Transcripts > 1000 bp annotated using KEGG*	21,040	21,126	19,210	18,646
**Total protein sequences**	**85,234**	**91,301**	**75,426**	**79,234**
*Proteins annotated using eggNOG: eggNOG annotations*	52,688	56,090	50,413	50,338
*Proteins annotated using eggNOG: KO annotations*	26,011	28,187	25,244	24,894
*Proteins annotated using Pfam-A*	44,390	46,868	42,244	42,258

## Data Availability

The datasets presented in this article will be made available by the authors for research purposes. Requests to access the datasets should be directed to the corresponding author (Uljana Hesse; uhesse@uwc.ac.za).

## Supplementary Materials

The following are available online at https://www.mdpi.com/2673-6284/9/4/19/s1, Figure S1: Distribution of the collected rooibos ecotypes sampled for this study, Table S1: Number of overlapping orthogroups predicted by OrthoFinder.
