# Supplementary material for: Transcriptomics of the Rooibos (Aspalathus linearis) Species Complex
Source: BioTech (Basel). 2020 Sep 23;9(4):19. doi: 10.3390/biotech9040019 (PMC9258316; doi:10.3390/biotech9040019)
Supplement: Supplementary file 1 [file biotech-09-00019-s001.pdf]

Supplementary Materials: Transcriptomics of the Rooibos (*Aspalathus linearis*) Species Complex

Emily A Stander [1], Wesley Williams [1,2], Yamkela Mgwatyu [1], Peter van Heusden [1], Fanie Rautenbach [3], Jeanine Marnewick [3], Marilize le Roes-Hill [3], Uljana Hesse [1,2,4,*]

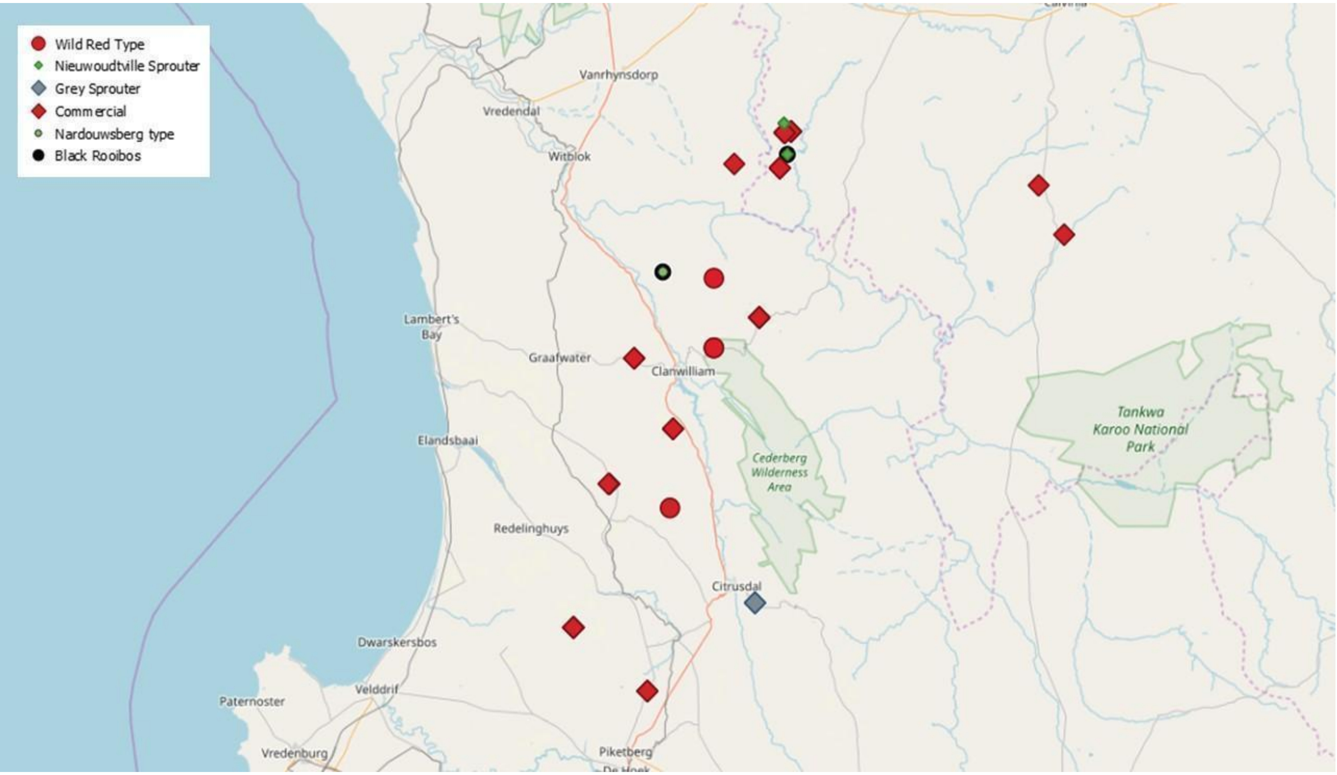

Supplemenatry Figure S1: Distribution of the collected rooibos ecotypes sampled for this study.

Supplemenatry Table S1: Number of overlapping orthogroups predicted by OrthoFinder.

	**number of sequences**	**A**	**B**	**C**	**D**	***L. angustifolius***	***L. japonicus***	***M. truncatula***	***A. thaliana***	***O. sativa***
**A**	85234	**29535**	20486	19144	19629	12744	13471	13333	11429	10943
**B**	91301	20486	**28650**	19545	19755	12819	13546	13439	11485	10992
**C**	74767	19144	19545	**26334**	18968	12823	13434	13360	11489	10937
**D**	79234	19629	19755	18968	**26912**	12604	13296	13187	11357	10873
L. angustifolius	33076	12744	12819	12823	12604	**14308**	12712	13185	11646	10777
L. japonicus	48105	13471	13546	13434	13296	12712	**16268**	13719	11564	10932
M. truncatula	57585	13333	13439	13360	13187	13185	13719	**15828**	11987	11229
A. thaliana	35386	11429	11485	11489	11357	11646	11564	11987	**12665**	10685
O. sativa	66338	10943	10992	10937	10873	10777	10932	11229	10685	**12526**
